# Glycyrrhizin regulates rat TMJOA progression by inhibiting the HMGB1‐RAGE/TLR4‐NF‐κB/AKT pathway

**DOI:** 10.1111/jcmm.17149

**Published:** 2021-12-24

**Authors:** Zhihui Hu, Mian Xiao, Hengxing Cai, Wei Li, Wei Fang, Xing Long

**Affiliations:** ^1^ The State Key Laboratory Breeding Base of Basic Science of Stomatology (Hubei‐MOST) & Key Laboratory of Oral Biomedicine Ministry of Education School and Hospital of Stomatology Wuhan University Wuhan China; ^2^ Affiliated Stomatological Hospital of Nanchang University Nanchang China; ^3^ Department of Oral and Maxillofacial Surgery School and Hospital of Stomatology Wuhan University Wuhan China

**Keywords:** glycyrrhizin, HMGB1, RAGE, temporomandibular joint osteoarthritis, TLR4

## Abstract

To investigate the role of glycyrrhizin on the progression of temporomandibular joint osteoarthritis (TMJOA) and the underlying mechanism by regulation of the high‐mobility group box 1 (HMGB1) receptor for advanced glycation end products (RAGE)/toll‐like receptor 4 (TLR4)‐nuclear factor kappa B (NF‐κB)/protein kinase B (AKT) pathway. After a rat model of TMJOA was built by intra‐articular injection of monosodium iodoacetate, glycyrrhizin was intragastrically administered at low concentration (20 mg/kg) or high concentration (50 mg/kg). Micro‐computed tomography, histological and immunohistochemical analysis were used to reveal the progression of TMJOA. Rat TMJ chondrocytes and disc cells were cultured in inflammatory condition with different doses of glycyrrhizin. Western blot was used to evaluate the effect of glycyrrhizin on the HMGB1‐RAGE/TLR4‐NF‐κB/AKT pathway. Administration of glycyrrhizin alleviated cartilage degeneration, lowered the levels of inflammatory and catabolic mediators and reduced the production of HMGB1, RAGE and TLR4 in TMJOA animal model. Increased production of RAGE and TLR4, and activated intracellular NF‐κB and/or AKT signalling pathways in chondrocytes and disc cells were found in inflammatory condition. Upon activation, matrix metalloprotease‐3 and interleukin‐6 were upregulated. Glycyrrhizin inhibited not only HMGB1 release but also RAGE and TLR4 in inflammatory condition. Glycyrrhizin alleviated the pathological changes of TMJOA by regulating the HMGB1‐RAGE/TLR4‐NF‐kB/AKT signalling pathway. This study revealed the potential of glycyrrhizin as a novel therapeutic drug to suppress TMJ cartilage degradation.

## 1 INTRODUCTION

Temporomandibular joint osteoarthritis (TMJOA) is a degenerative joint disease characterized by progressive cartilage degradation, subchondral bone remodelling, chronic synovitis and disc perforation, which lead to joint pain, popping and limited mouth opening.^1^, ^2^ TMJOA therapy is directed at relieving symptoms, decelerating the progress of the disease and restoring TMJ function. Conservative treatments for TMJOA are preferred including physical therapies, occlusal splints and nonsteroidal anti‐inflammatory drugs.^3^ In view of the limited understanding of its pathogenesis and the low healing potential of avascular cartilage, there is no effective conservative treatment to restore the structures of the TMJ with progressive OA.^4^

TMJOA is generally considered a “low‐inflammatory arthritic condition”, accompanied by an increase in a variety of inflammatory cytokines.^5^ The occurrence and development of TMJOA are closely related to an inflamed TMJ.^6^ Recently, high‐mobility group box 1 (HMGB1), a highly conserved nonhistone nuclear protein belonging to the “high‐mobility group” protein family, was found to be closely associated with the pathogenesis of TMJOA.^7^, ^8^ It was shown that the expression of HMGB1 was increased in inflamed synovium and disc of human TMJOA, and it regulated the angiogenesis of perforated disc cells.^8^, ^9^ In TMJ cartilage, HMGB1 translocated from the nucleus to the cytoplasm after interleukin‐1β (IL‐1β) incubation and is released into the extracellular matrix (ECM) in an inflamed TMJOA animal model.^10^

Glycyrrhizin is a natural triterpene glycoconjugate derived from the root of licorice.^11^ It has been described by various pharmacological properties, including antiviral, anti‐inflammatory, antitumor and hepatoprotective activities, and it is commonly used in chronic hepatitis treatment.^12^ Glycyrrhizin has been shown to bind HMGB1 and counteract its chemokine‐ and cytokine‐mediated inflammatory response.^11^ It has been reported that glycyrrhizin reduces the production of OA‐related biomarkers induced by HMGB1 in knee chondrocytes, such as matrix metalloprotease‐3 (MMP‐3) and MMP‐13.^13^ However, the role of glycyrrhizin in TMJOA is not clear.

HMGB1 binds to cell surface receptors, such as toll‐like receptor 4 (TLR4) and receptor for advanced glycation end products (RAGE), and then activates multiple intracellular signalling pathways to promote the expression and release of inflammatory cytokines in various cells.^14^ TLRs and RAGE are critical pattern recognition receptors expressed in OA cartilage and surrounding tissue, recognizing various damage‐associated molecular patterns, including HMGB1.^15^ Stimulation of RAGE and TLR4 has been shown to activate the nuclear factor kappa B (NF‐κB) p65 pathway, which triggers the upregulation of proinflammatory cytokines in the colon and synovium,^16^, ^17^ and activates the protein kinase B (AKT) signalling pathway in the liver.^18^

Herein, this study aimed to investigate the effect of glycyrrhizin on the pathogenesis of TMJOA, and its regulation of the HMGB1‐RAGE/TLR4‐NF‐κB/AKT pathway.

## 2 MATERIALS AND METHODS

### 2.1 Rats

Male Sprague‐Dawley (SD) rats were purchased from Hubei Provincial Centers for Disease Control and Prevention and housed in a specific pathogen‐free laboratory for at least 7 days before use. All experimental procedures were approved by the Ethics Committee for Animal Research, School and Hospital of Stomatology, Wuhan University.

### 2.2 Glycyrrhizin for treatment of TMJOA model

Rat TMJOA induced by monosodium iodoacetate (MIA) (57858; Sigma) was used to evaluate the therapeutic effects of glycyrrhizin (50531; Sigma). Forty‐eight 8‐week‐old SD rats were randomly divided into four groups: control, MIA, MIA+glycyrrhizin (20 mg/kg) and MIA+glycyrrhizin (50 mg/kg) (*n* = 6). The reagent (1 mg MIA dissolved in 50 μl saline) was bilaterally injected into the upper compartment of the TMJ to induce TMJOA. Glycyrrhizin was intragastrically administered at low concentration (20 mg/kg) or high concentration (50 mg/kg) after intra‐articular injection of MIA, and then once a day for 2 or 4 weeks. After 2 weeks, the rats were sacrificed and subjected to histological and immunohistochemical analysis. After 4 weeks, the rats were sacrificed and subjected to micro‐computed tomography (micro‐CT) examination.

### 2.3 Micro‐CT

The joints were scanned by a micro‐CT system (SkyScan 1176; Bruker Corp) at 385 μA, 65 kV and a thickness of 9 μm per slice. Radiographs were reconstructed using NRecon and analysed by CTAn for the relative parameters including bone volume fraction (BV/TV), trabecular thickness (Tb. Th), trabecular separation (Tb. Sp) and trabecular number (Tb. N). 3D images were obtained for morphological assessment with CTvox software.

### 2.4 Histological analysis

The TMJ specimens were fixed in 4% paraformaldehyde, decalcified in 10% ethylenediaminetetraacetic acid and embedded in paraffin after gradient dehydration. The paraffin blocks were sectioned at a thickness of 5 μm, and the sections were deparaffinized in xylene and hydrated with graded ethanol. The sections were stained with the Haematoxylin and Eosin (HE) Staining Kit (G1005; Servicebio) and Safranine O‐Fast Green Staining Kit (CR2012068; Servicebio) according to the manufacturer's protocol. Proteoglycan changes in the cartilage matrix were detected using safranin O staining. The modified Mankin score system was used for assessment of TMJOA.^19^, ^20^ Histological evaluation was described in four aspects: cartilage structure (0–3 points) (smooth non‐eroded cartilage, score 0; rough non‐eroded cartilage, score 1; superficial fibrillation, score 2; separation of uncalcified from calcified cartilage, score 3); pericellular matrix staining (0–2 points) (normal, score 0; slightly enhanced, score 1; intensely enhanced, score 2); spatial arrangements of chondrocytes (0–3 points) (normal, score 0; diffuse hypercellularity, score 1; clustering, score 2; hypocellularity, score 3); background matrix staining (0–3 points) (normal, score 0; slight increased or decreased, score 1; severe increase or decrease, score 2; no staining, score 3).

### 2.5 Immunohistochemistry

The sections were microwave antigen‐retrieved in citrate solution. For immunohistochemistry (IHC), commercial IHC kits (KIT‐9707; Maixin) were used according to the manufacturer's specifications. Endogenous peroxidase activity and nonspecific binding were blocked. Then the sections were incubated with primary antibodies against HMGB1 (1:300; EPR3507; Abcam), RAGE (1:50; 16346‐1‐AP; Proteintech), TLR4 (1:400; GB11519; Servicebio), MMP‐3 (1:200; 17873‐1‐AP, Proteintech), MMP‐9 (1:400; GB11132; Servicebio), MMP‐13 (1:300; GB11247; Servicebio), IL‐1β (1:100; ab9722; Abcam), interleukin‐6 (IL‐6) (1:200; GB11117; Servicebio) and TNF‐α (1:100; ab6671; Abcam) overnight at 4°C in a humidified chamber. The sections were washed with phosphate‐buffered saline (PBS) and incubated with secondary antibodies. Finally, the sections were coloured by reacting with 3,3‐diaminobenzidine (DAB‐0031; Maixin). Haematoxylin was used for counterstaining the structure. Average optical density and positive cells count was determined by researchers who were blinded to the groups using ImageJ.

### 2.6 Culture of TMJ chondrocytes and disc cells

TMJ cartilage and articular disc tissue were harvested from 4‐week‐old SD rats. After cutting into small pieces, the tissue was digested with 0.25% trypsin (SH30042.01; HyClone) for 20 min. Then, cartilage tissue was digested in DMEM (SH30022.01B; HyClone) containing 0.1% collagenase type I and 0.1% collagenase type II for 1 h, and the disc tissue was digested for 45 min. Digested chondrocytes and disc cells were collected and resuspended in DMEM containing 20% fetal bovine serum (10099; Gibco) after centrifugation. Finally, the cells were cultured in an atmosphere of 5% CO_2_.

### 2.7 Conditional culture

Chondrocytes and disc cells within the third passage were stimulated with 100 ng/ml HMGB1 (H4652; Sigma) or 10 ng/ml IL‐1β (400‐01B; Peprotech) in the presence or absence of inhibitors. Glycyrrhizin, a HMGB1 inhibitor, was used to pretreat cells at different concentrations (0.1–200 μM/ml) for 1 h, followed by incubation with IL‐1β (10 ng/ml) for 24 h. TAK‐242 (S7455; Selleck) is a TLR4 inhibitor, and FPS‐ZM1 (S8185; Selleck) is a RAGE inhibitor; both were used to pretreat cells at different concentrations (5–40 μM/ml) 1 h before HMGB1 stimulation. In chondrocytes pretreated with or without TAK‐242 (10 μM/ml) and/or FPS‐ZM1 (40 μM/ml), HMGB1 (100 ng/ml) was applied for 24 h. The disc cells were pretreated with or without TAK‐242 (5 μM/ml) and/or FPS‐ZM1 (20 μM/ml). Then, the culture supernatant was collected, and the concentration of IL‐6 was detected using a commercial ELISA kit (CRE0005; 4A Biotech).

### 2.8 Western blotting

Conditional cultured chondrocytes and disc cells were washed with PBS and lysed with RIPA buffer containing a proteinase and phosphatase inhibitor. The cell lysates were sonicated, followed by heating and adding a loading buffer. Processed protein samples were loaded onto 10% SDS‐PAGE gels, separated by electrophoresis, and then electrotransferred to PVDF membranes. The blotting membranes were blocked with 5% nonfat milk for 1 h at room temperature and incubated overnight at 4°C with primary antibodies, including HMGB1 (1:1000; EPR3507; Abcam), RAGE (1:1000; EPR21171; Abcam), TLR4 (1:1000; 19811‐1‐AP; Proteintech), MMP‐3 (1:10000; 66338‐1‐Ig; Proteintech), NF‐κB p65 (1:1000; 8242; CST), phospho‐NF‐κB p65 (1:1000; 3033; CST), AKT (1:1000; 4691; CST), phospho‐AKT (1:1000; AP0140; ABclonal) and GAPDH (1:5000; GB11002; Servicebio). The proteins were detected with horseradish peroxidase‐conjugated secondary antibodies and visualized using Western Bright ECL (K‐12045‐D10; Advansta). The blotted protein bands were quantitatively analysed using ImageJ software.

### 2.9 Statistical analysis

All statistical analyses were performed using GraphPad Prism 8.0.1 software. The Shapiro‐Wilk test was performed to determine the suitability test for the normality of the data, and the *F* test was performed to determine whether the population variances were equal. Student's *t* test was used in comparison with datasets obeying normality distribution; otherwise, a nonparametric Mann‐Whitney *U* test was performed. All data were obtained from at least three independent experiments (*n* ≥ 3) and are presented as the mean ± SD. For all tests, *p* < 0.05 was considered statistically significant.

## 3 RESULTS

### 3.1 Glycyrrhizin alleviated cartilage degeneration in experimental TMJOA in rats

**FIGURE 1:**
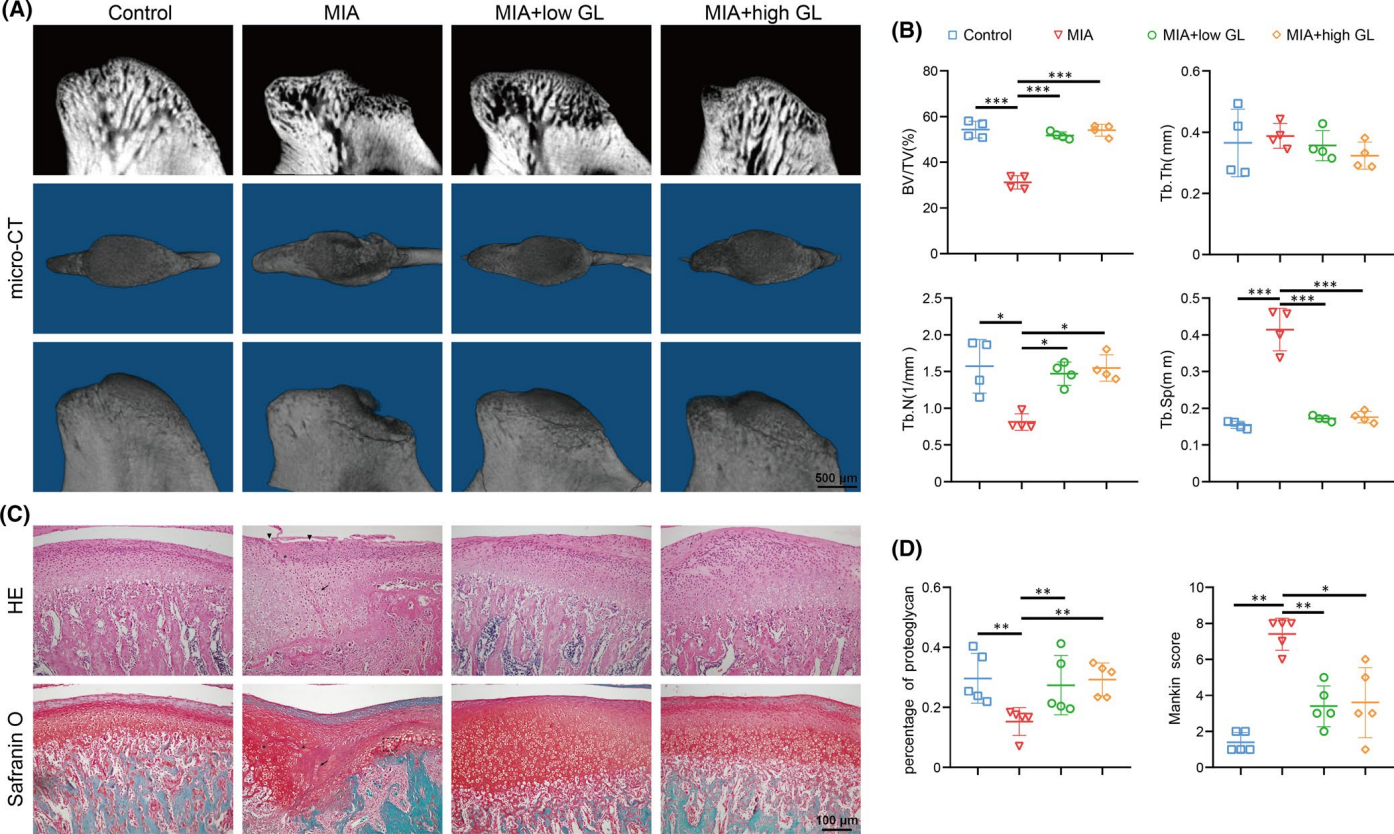
Glycyrrhizin (GL) attenuated bone deterioration and improved cartilage structure in temporomandibular joint osteoarthritis rats. Glycyrrhizin was intragastrically administrated at low concentration (20 mg/kg) and high concentration (50 mg/kg) after intraarticular injection of monosodium iodoacetate (MIA) and then once daily afterwards for 4 or 2 weeks. (A) Representative three‐dimensional reconstruction and sagittal view for four groups of TMJ condyles by micro–computed tomography (micro‐CT). (B) Statistical analysis of relative bone deterioration parameters: bone volume fraction (BV/TV), trabecular thickness (Tb. Th), trabecular number (Tb. N) and trabecular separation (Tb. Sp). (C) HE staining and safranin O staining showed the changes of cartilage structure and matrix proteoglycan. Triangles = fissures; asterisks = acellular regions (fibrosis); black arrows = cell cluster; square = enhanced pericellular matrix staining. (D) The percentage of proteoglycan decreased in the MIA group, whereas glycyrrhizin administration ameliorated the reduction in cartilage proteoglycans induced by MIA. Mankin scores were obtained according to safranin O and HE staining. Data are presented by mean ± SD (*n* ≥ 4). *p < 0.05, **p < 0.01, ***p < 0.001

Based on micro‐CT examination, there was severe bone destruction and discontinuous subchondral bone in the anterior slope of the condyle in the MIA group, whereas there were reduced lesions and improved appearance in the glycyrrhizin groups (Figure 1A). Relative micro‐CT parameters including bone volume fraction (BV/TV), trabecular number (Tb. N) and trabecular separation (Tb. Sp) were consistent with the morphological assessment, although there was no difference in trabecular thickness (Tb. Th) (Figure 1B). Safranin O staining showed that a reduction in proteoglycans occurred in the cartilage of the MIA group, compared with that of the control, which maintained abundant and regular proteoglycans (Figure 1C). It should be noted that glycyrrhizin ameliorated the lessening of cartilage proteoglycans induced by MIA (Figure 1C,D). HE staining showed the improvement of cartilage structure and cellularity after glycyrrhizin administration (Figure 1C). According to the Mankin scoring system, a significant difference in Mankin scores was observed in the control, MIA and MIA+glycyrrhizin groups (Figure 1C,D). The score of the MIA group was higher than that of control and glycyrrhizin treatment groups. The itemized Mankin scores for each category showed similar changes (Figure S1). Glycyrrhizin treatment showed obvious improvement on structural integrity, as well as cartilage matrix. These results collectively suggested that glycyrrhizin protected cartilage from degradation.

### 3.2 Glycyrrhizin alleviated inflammation and matrix degeneration of osteoarthritic cartilage

**FIGURE 2:**
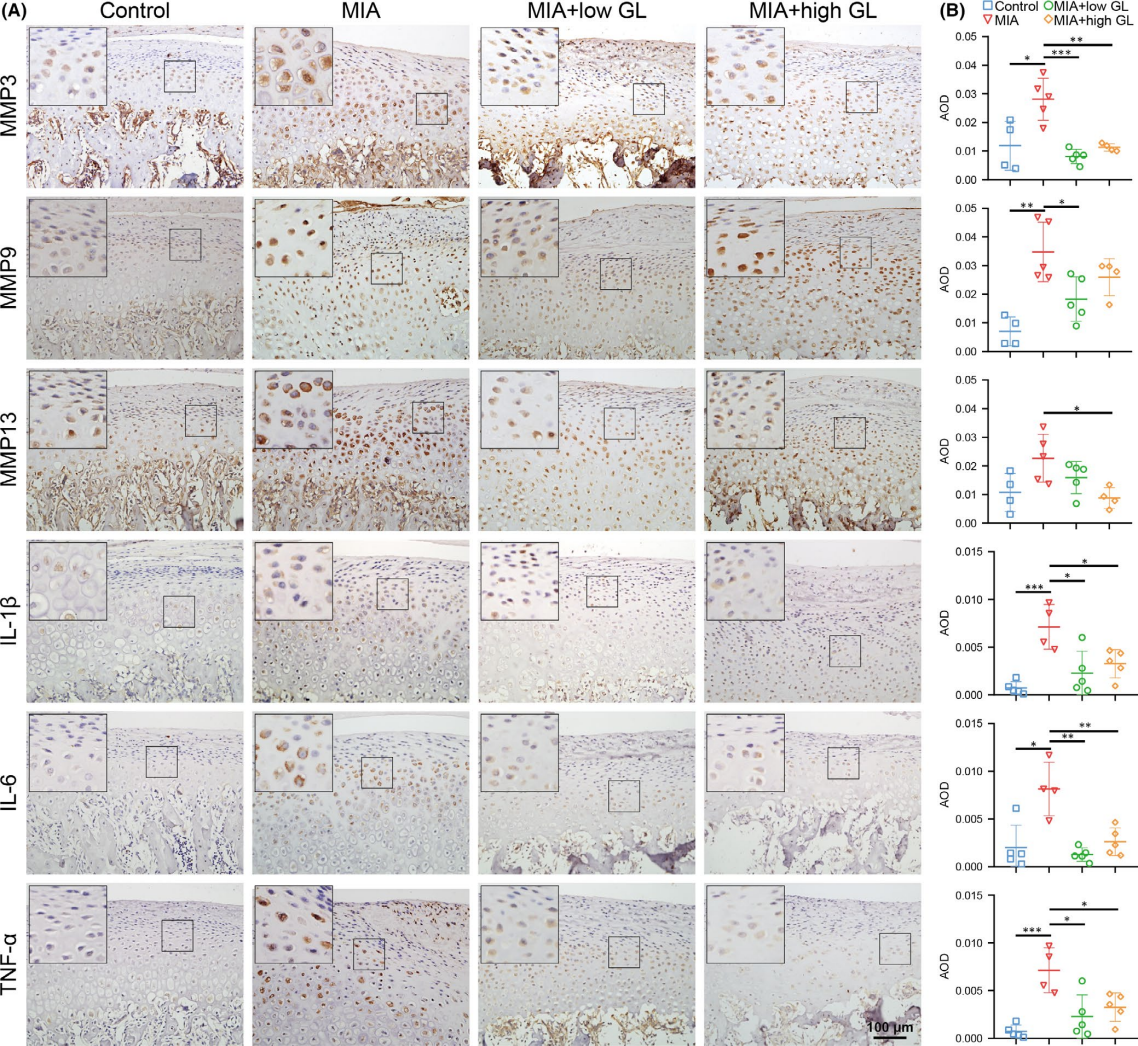
Glycyrrhizin reduced cartilage inflammation and degeneration in temporomandibular joint osteoarthritis rats. Glycyrrhizin was intragastrically administrated at low concentration (20 mg/kg) and high concentration (50 mg/kg) after intraarticular injection of monosodium iodoacetate (MIA) and then once daily afterwards for 2 weeks. (A) Immunohistochemistry staining was used to detect MMP‐3, MMP‐9, MMP‐13, IL‐1β, IL‐6 and TNF‐α protein expression in TMJ cartilage. (B) Average optical density (AOD) was measured to show the positive staining. Data are presented by mean ± SD (*n* = 5). *p < 0.05, **p < 0.01, ***p < 0.001

The expression of proinflammatory mediators (IL‐1β, IL‐6 and TNF‐α) and catabolic mediators (MMP‐3, MMP‐9 and MMP‐13) in TMJ cartilage was detected. Compared with the control group, the expression of IL‐1β, IL‐6, TNF‐α, MMP‐3, MMP‐9 and MMP‐13 in the MIA group increased significantly, and all decreased after treatment with glycyrrhizin. Different concentrations of glycyrrhizin have similar effects in TMJOA cartilage (Figure 2A,B). This indicated that MIA caused inflammation and matrix degradation in TMJ cartilage, while glycyrrhizin exerted a protective effect by reducing the protein expression of aforementioned mediators.

### 3.3 Glycyrrhizin reduced the expression of HMGB1, RAGE and TLR4 in TMJOA cartilage

**FIGURE 3:**
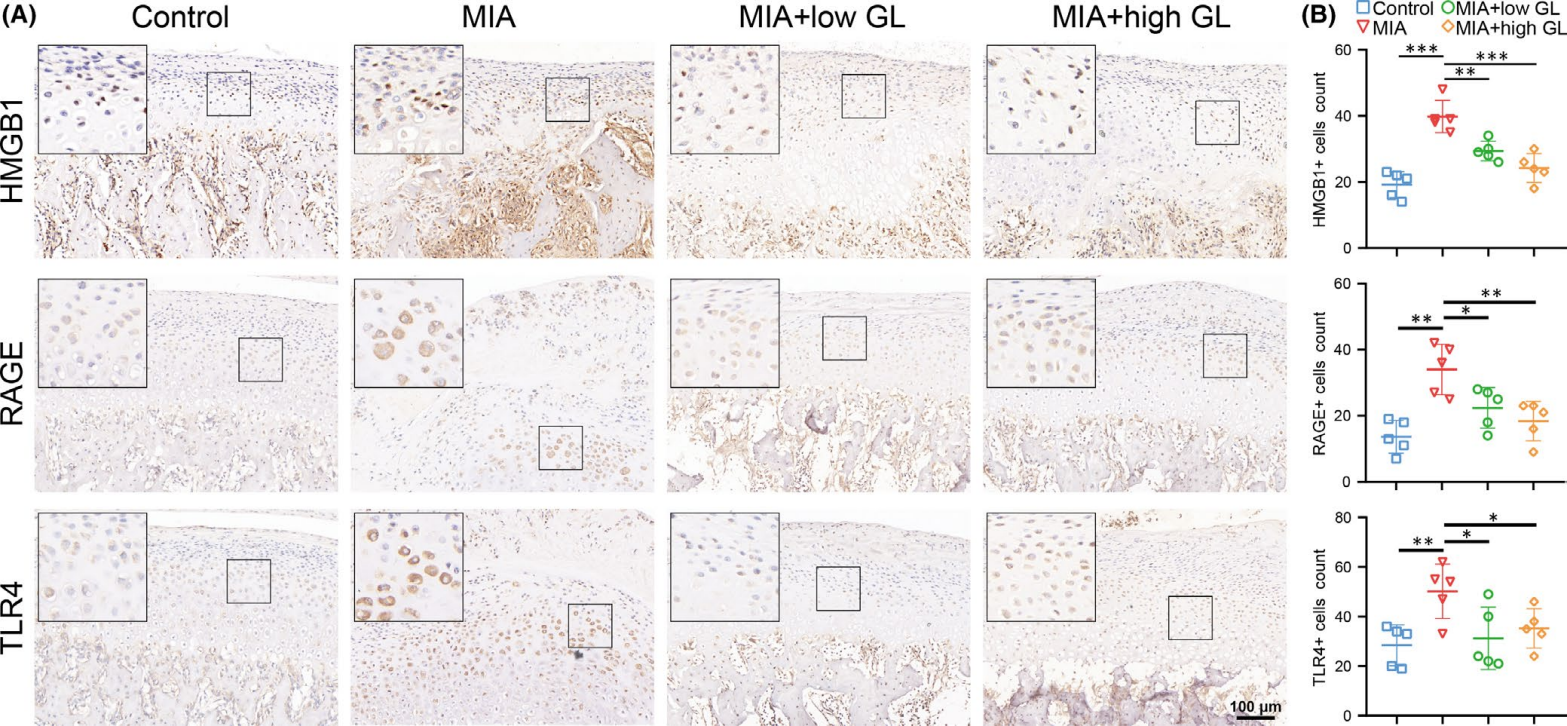
Glycyrrhizin downregulated the expression of high‐mobility group box 1 (HMGB1), RAGE and TLR4 in temporomandibular joint osteoarthritis cartilage. Glycyrrhizin was administered intragastrically at low concentration (20 mg/kg) and high concentration (50 mg/kg) after intraarticular injection of monosodium iodoacetate (MIA) and then once daily afterward for 2 weeks. (A) Immunohistochemistry staining was used to detect HMGB1, RAGE and TLR4 protein expression in rat TMJ cartilage. (B) The positive cells were counted to show the positive staining. Data are presented by mean ± SD (*n* = 5). *p < 0.05, **p < 0.01, ***p < 0.001

HMGB1‐positive staining was significantly increased in osteoarthritic cartilage compared with normal cartilage. There was also a significant increase in the number of RAGE and TLR4 positive cells in the MIA group (Figure 3A). The expression of RAGE was mainly concentrated in the flattened chondrocyte layer, while TLR4 was expressed throughout the whole‐layer cartilage. Under the administration of glycyrrhizin at different concentrations, the expression of HMGB1 and its receptors decreased in condylar cartilage (Figure 3B).

### 3.4 Glycyrrhizin inhibited the expression of HMGB1 in vitro

**FIGURE 4:**
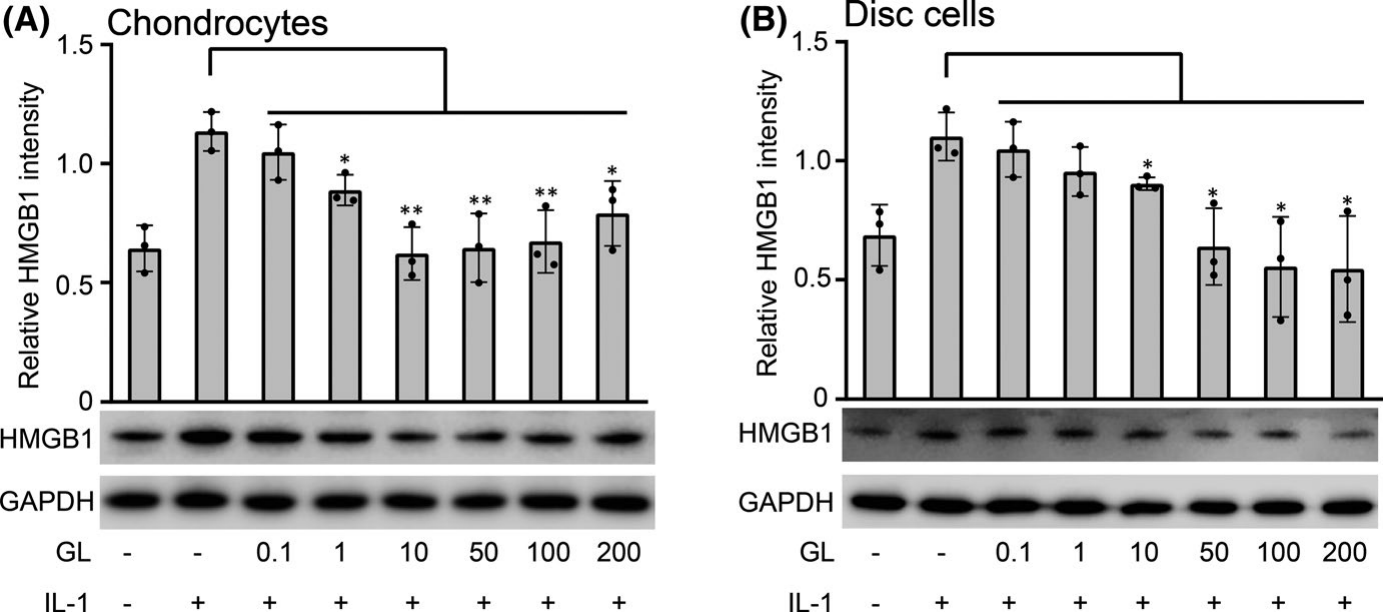
Glycyrrhizin, an high‐mobility group box 1 (HMGB1) inhibitor, inhibited the expression of HMGB1 induced by IL‐1β. (A) Chondrocytes were pretreated with different doses of glycyrrhizin (0.1–200 μM/ml) for 1 h, followed by incubation with IL‐1β (10 ng/ml) for 24 h. Then, HMGB1 protein expression was assessed by Western blot (WB) analysis. (B) Disc cells were pretreated with different doses of glycyrrhizin (0.1–200 μM/ml) for 1 h, followed by incubation with IL‐1β (10 ng/ml) for 24 h. Then, HMGB1 protein expression was assessed by WB analysis. GAPDH served as the loading control. Data are presented by mean ± SD (*n* = 3). *p < 0.05, **p < 0.01

In chondrocytes, IL‐1β stimulation increased the expression of HMGB1 (Figure 4A). Similar results were obtained in disc cells (Figure 4B). Before IL‐1β stimulation, chondrocytes and disc cells were pretreated with different concentrations of glycyrrhizin. As the concentration of glycyrrhizin increased, the expression of HMGB1 in chondrocytes was inhibited, and the inhibitory effect was strongest when the concentration of glycyrrhizin was 10 μM/ml (Figure 4A). In disc cells, the expression of HMGB1 gradually decreased as the concentration of glycyrrhizin increased (Figure 4B).

### 3.5 HMGB1 activated the RAGE/TLR4‐NF‐κB p65/AKT signalling pathways to upregulate MMP‐3 and IL‐6

**FIGURE 5:**
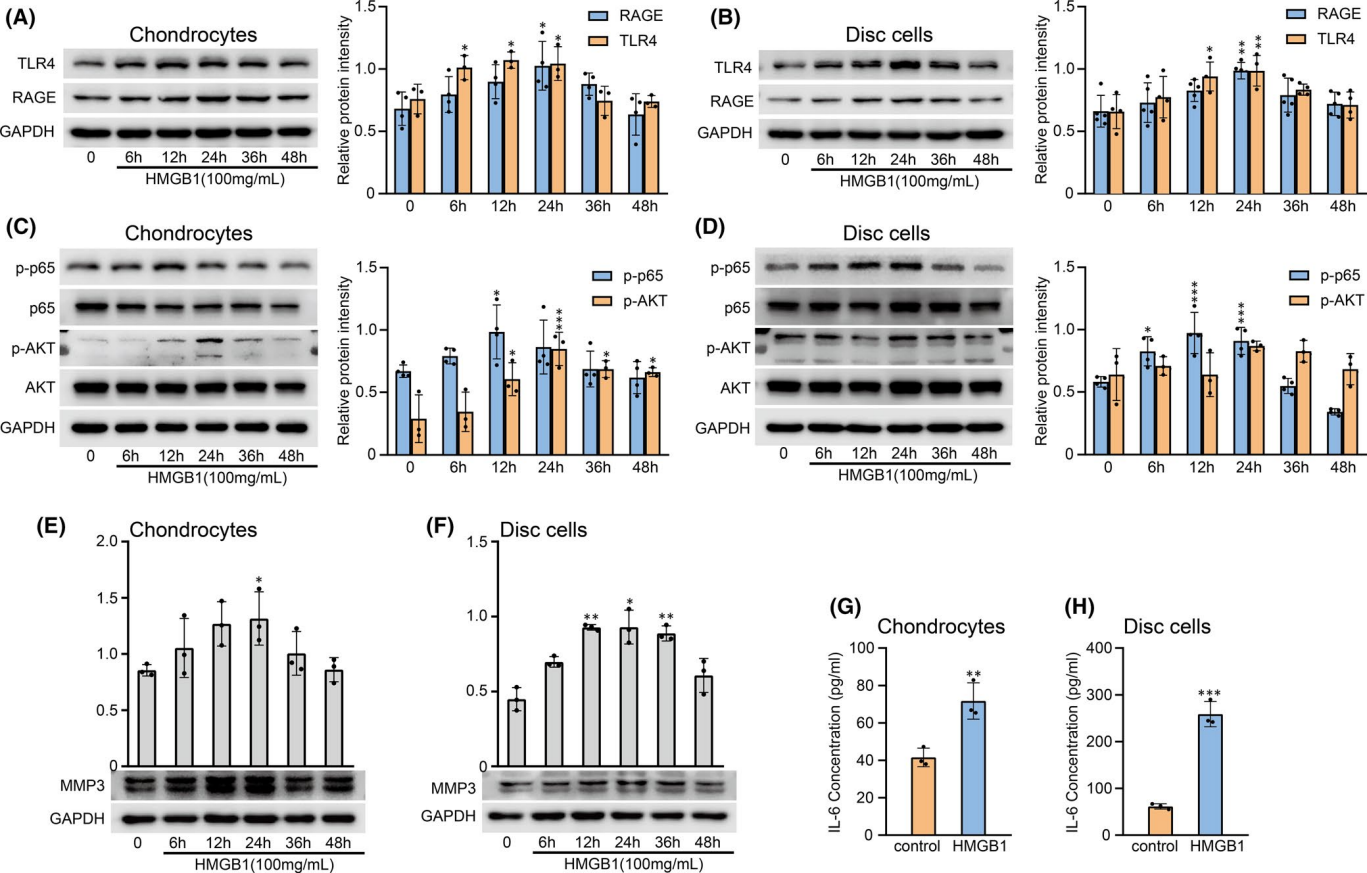
High‐mobility group box 1 (HMGB1) upregulated the expression of RAGE, TLR4, NF‐κB, AKT, MMP3 and IL‐6 in TMJ chondrocytes and disc cells. (A, C, E) Rat primary cultured chondrocytes were treated with HMGB1 (100 ng/ml) for various times (0–48 h), and WB analysis was performed to determine RAGE, TLR4, p‐p65, p‐AKT and MMP3 protein expression. (B, D, F) Disc cells were treated with HMGB1 (100 ng/ml) for various times (0–48 h), and WB analysis was performed to determine RAGE, TLR4, p‐p65, p‐AKT and MMP3 protein expression. (G, H) The expression of IL‐6 was detected in the culture supernatant of chondrocytes and disc cells stimulated with HMGB1 for 24 h. Glyceladehyde‐3‐phosphate dehydrogenase (GAPDH) served as the loading control. Data are presented by mean ± SD (*n* = 3). *p < 0.05, **p < 0.01, ***p < 0.001

Previous research showed that the effect of promoting inflammation was most apparent when the concentration of HMGB1 was 100 ng/ml.^9^ HMGB1 stimulation of chondrocytes increased RAGE and TLR4 protein levels in a time‐dependent manner (Figure 5A). RAGE protein expression increased 6 h after HMGB1 stimulation and reached a maximal level at 24 h. TLR4 protein levels were markedly increased at 12 h and decreased to baseline at 36 h. Similar to chondrocytes, HMGB1 upregulated RAGE and TLR4 protein expression in disc cells (Figure 5B). TLR4 protein expression reached a maximal level at 24 h and returned to baseline levels after 48 h.

To investigate the potential intracellular signalling pathways, we assessed the protein levels of p65, AKT and their respective active forms (p‐p65 and p‐AKT) in chondrocytes and disc cells treated with HMGB1 for different times (Figure 5C,D). Chondrocytes increased the protein levels of p‐p65 and p‐AKT in a time‐dependent manner, but no significant difference in p65 and AKT was detected (Figure 5C). The expression of p‐p65 peaked at 12 h, while p‐AKT peaked at 24 h. In addition, disc cells only increased p‐p65 protein expression and reached a peak at 12 h, while no significant difference was detected between p65, AKT and p‐AKT (Figure 5D). The above results indicated that the NF‐κB p65 pathway was activated by HMGB1 in both cell types, while the AKT pathway was activated only in chondrocytes.

After that, in both chondrocytes and disc cells, MMP‐3 protein was gradually increased within 24 h after treatment and then decreased (Figure 5E,F). The expression of IL‐6 was also increased in the culture supernatant of chondrocytes and disc cells stimulated with HMGB1 for 24 h (Figure 5G,H). The above results indicated that HMGB1 is a proinflammatory and catabolic regulator in TMJ tissue.

### 3.6 Inhibition of RAGE and TLR4 reduced the expression of MMP‐3 and IL‐6

**FIGURE 6:**
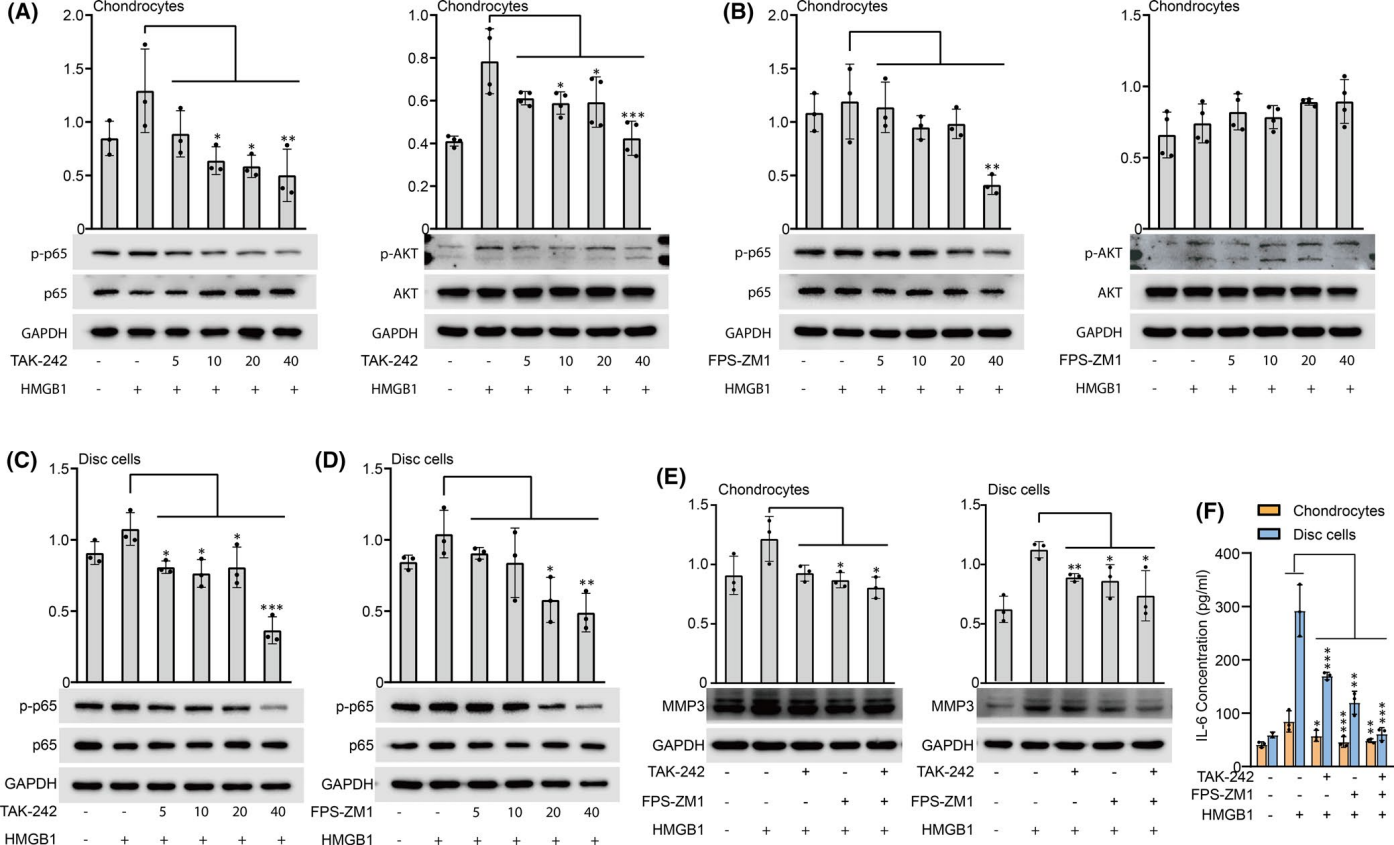
RAGE and TLR4 inhibitors reduced the expression of signalling molecules and inflammatory factors. A TLR4 inhibitor (TAK‐242) and RAGE inhibitor (FPS‐ZM1) were used and the concentration gradient was 5–40 μM/ml. (A, B) Chondrocytes were pretreated with different doses of TAK‐242 and FPS‐ZM1 for 1 h, followed by incubation with high‐mobility group box 1 (HMGB1) (100 ng/ml) for 24 h, and then p‐p65 and p‐AKT protein expression was assessed by WB analysis. (C, D) Disc cells were pretreated with different doses of TAK‐242 and FPS‐ZM1 for 1 h, followed by incubation with HMGB1 (100 ng/ml) for 24 h, and p‐p65 protein expression was assessed by WB analysis. (E) In chondrocytes and disc cells pretreated with TAK‐242 (10 μM/ml) and/or FPS‐ZM1 (40 μM/ml) for 1 h, MMP3 protein expression was examined after stimulation with HMGB1 for 24 h. (F) The expression of IL‐6 was examined in the culture supernatant of chondrocytes and disc cells pretreated with inhibitors after stimulation with HMGB1 for 24 h. GAPDH served as the loading control. Data are presented by mean ± SD (*n* = 3). *p < 0.05, **p < 0.01, ***p < 0.001

We examined whether TLR4 and RAGE inhibitors can prevent the increased expression of signalling molecules and proinflammatory cytokines. Here, a TLR4 inhibitor (TAK‐242) and RAGE inhibitor (FPS‐ZM1) were used to pretreat cells before HMGB1 stimulation. In chondrocytes, TAK‐242 reduced the production of p‐p65 and p‐AKT, while FPS‐ZM1 only caused a decrease in p‐p65 (Figure 6A,B). In disc cells, TAK‐242 and FPS‐ZM1 had the same inhibitory effect, reducing the production of p‐p65 induced by HMGB1 (Figure 6C,D). After pretreatment with TAK‐242 and/or FPS‐ZM1, HMGB1‐induced MMP‐3 production was reduced in both chondrocytes and disc cells (Figure 6E). Similar results were observed in the secretion of IL‐6 protein in the cell culture supernatant (Figure 6F). This indicated that inhibition of RAGE and TLR4 could block the inflammatory signal transduction caused by HMGB1.

### 3.7 Glycyrrhizin inhibited the expression of RAGE and TLR4 in chondrocytes and disc cells

**FIGURE 7:**
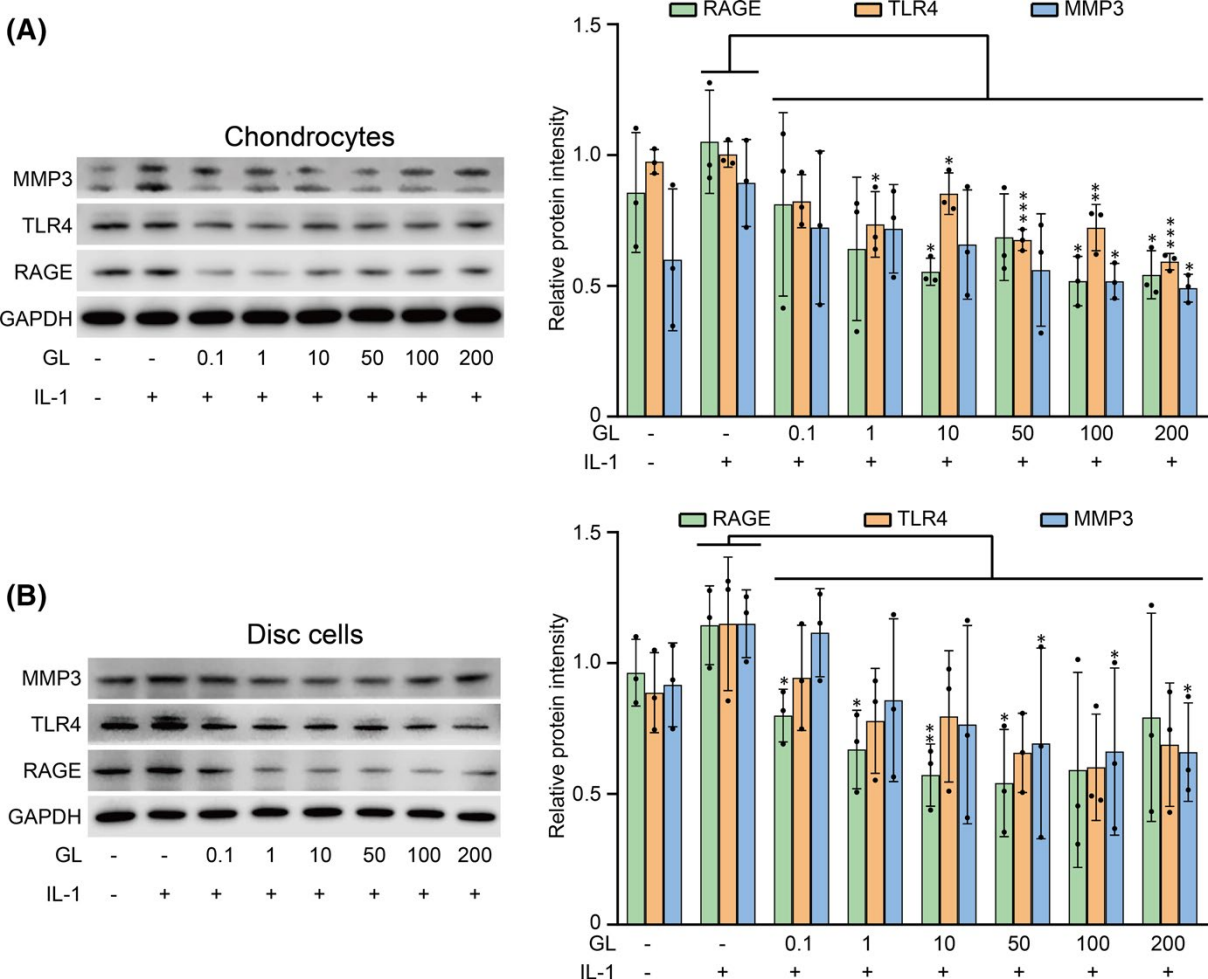
Glycyrrhizin inhibited the expression of RAGE, TLR4 and MMP3. (A) Chondrocytes were pretreated with different doses of glycyrrhizin (0.1–200 μM/ml) for 1 h, followed by incubation with IL‐1β (10 ng/ml) for 24 h. Then, RAGE, TLR4 and MMP3 protein expression was assessed by WB analysis. (B) Disc cells were pretreated with different doses of glycyrrhizin (0.1–200 μM/ml) for 1 h, followed by incubation with IL‐1β (10 ng/ml) for 24 h. Then, RAGE, TLR4 and MMP3 protein expression was assessed by WB analysis. GAPDH served as the loading control. Data are presented by mean ± SD (*n* = 3). *p < 0.05, **p < 0.01, ***p < 0.001

Glycyrrhizin was used to pretreat cells at different concentrations before IL‐1β stimulation. IL‐1β increased the expression of RAGE and TLR4 in chondrocytes and disc cells (Figure 7A,B). However, glycyrrhizin reversed the effect of IL‐1β and decreased the expression of RAGE and TLR4 (Figure 7A,B). In chondrocytes and disc cells, the expression of MMP‐3 was also downregulated by glycyrrhizin (Figure 7A,B). This showed that glycyrrhizin could inhibit the inflammatory pathway activated by HMGB1.

## 4 DISCUSSION

Glycyrrhizin, the major constituent of licorice root, is identified by its anti‐inflammatory, anticancer and antioxidative effects.^21^ It possesses numerous pharmacological effects and has been shown to be effective in animal models of colitis, keratitis and brain injury.^22^, ^23^, ^24^ Most of the currently available TMJOA treatment lines are palliative and do not replace the degrading cartilage and subchondral bone. In this study, the efficacy of glycyrrhizin was evaluated as a therapeutic agent for experimental TMJOA in rats. The dose and the route of administration of glycyrrhizin were selected because of reported good bioavailability.^25^ Previous studies confirmed that MIA successfully induced TMJOA in animals and studied potential treatments for TMJ.^26^, ^27^ The etiology of TMJOA is complex, while the mechanism of MIA induced TMJOA is to disrupt chondrocytes glycolysis, resulting in the histological and morphological changes of the condyle cartilage, which are similar to the pathological manifestations of OA patients.^28^, ^29^ Glycyrrhizin attenuated the MIA‐induced manifestation of TMJOA in rats. Administration of glycyrrhizin alleviated bone destruction and promoted trabecular bone reconstruction; more obviously, the surface morphology of the condyle was improved and more continuous. In other words, both condylar cartilage and subchondral bone were protected by glycyrrhizin from OA‐related pathology. Additionally, glycyrrhizin has been suggested to inhibit the release of HMGB1 and bind to HMGB1 directly, resulting in inhibition of the proinflammatory cytokine‐like activity of this protein.^23^, ^30^, ^31^ In this study, after glycyrrhizin administration, the expression of HMGB1 was downregulated both in vitro and in vivo. Similar to this study, there was an improvement of inflammation by the significant reduction of HMGB1 and MMPs expression in rheumatoid arthritis after glycyrrhizin treatment.^32^

The proinflammatory activity of HMGB1 was first discovered in studies of sepsis, and subsequently, the importance of HMGB1 as a proinflammatory cytokine has been demonstrated in many inflammation‐associated diseases.^33^ Increased HMGB1 expression has been demonstrated in inflammatory joint diseases such as rheumatoid arthritis, osteoarthritis and synovitis.^9^, ^34^ It has been shown that the expression of HMGB1 is increased in inflamed synovium and perforated discs in TMJOA, indicating that HMGB1 expression is correlated with the development of TMJOA and promotes the inflammatory response.^8^, ^9^ It is also reported that the chondrocytes secrets HMGB1 to the ECM at the late stage of inflammation.^13^ HMGB1 is redistributed from the nucleus to cytoplasmic organelles and secreted by stimuli triggering lysosome exocytosis in monocytes.^35^ With the current understanding of “low‐inflammatory conditions” in TMJOA, the biological balance is compromised.^36^ This could be explained by the anti‐inflammatory defence mechanism's incapability to keep pace with inflammatory factor generation due to increased cytokine production. Consistent with this study, the expression of inflammatory factors IL‐1β and TNF‐α in cartilage is increased significantly in knee OA cartilage 7 weeks after MIA injection.^37^ HMGB1 serves as a signalling molecule involved in acute and chronic inflammation, for example, in sepsis and arthritis.^38^ In vivo experiments showed that glycyrrhizin reduced the expression of HMGB1, TNF‐α and IL‐1β in osteoarthritic cartilage, as reported in keratitis and necrotizing enterocolitis.^16^, ^22^ Glycyrrhizin suppressed the translocation and release of HMGB1 by inhibiting its phosphorylation.^39^ In this study, the expression of HMGB1 in TMJOA cartilage was decreased by glycyrrhizin treatment, accompanied by a decrease in RAGE and TLR4. It has been reported that glycyrrhizin blocks the interaction of HMGB1 and RAGE by binding with HMGB1.^23^ Another research shows that glycyrrhizin can inhibit TLR4 translocation to lipid rafts, which is involved in TLR4 signalling.^40^

It has been shown that exogenous HMGB1 has the same proinflammatory effect as native HMGB1 in many diseases.^14^ In vitro experiments of HMGB1‐incubated TMJ chondrocytes and disc cells, the expression of RAGE and TLR4 was upregulated. RAGE and TLR4 are the most characteristic cell surface receptors for proinflammatory response mediated by HMGB1.^41^ Studies have reported the role of RAGE in HMGB1‐induced inflammation, regeneration and autophagy and reported that TLR4 recognizes several danger signals, including HMGB1, to activate the innate immune system to prevent infection and injury.^42^ Consistent with this study, extracellular HMGB1 can stimulate RAGE expression in several cell types.^43^ However, another study showed that HMGB1 increased RAGE and TLR2 expression and had no effect on TLR4 protein in fibroblasts.^44^ Although it is unclear what accounts for these differences, they may be attributable to differences in the source and type of experimental cells. Though TLR4 are membrane receptors, other studies have described nuclear expression of TLR4 in several cell types,^45^, ^46^, ^47^ which was also observed in the cartilage of TMJOA in this study. But the precise implication of this is not understood. It is reported that TLR4 is colocalized with LPS in nucleus of lung cells, but it was not clear where the TLR4‐LPS complex was formed, in the cell surface or in the cytoplasm, or whether this complex was formed in the nucleus with pre‐existing TLR4.^48^

Stimulation of RAGE and TLR4 has been shown to activate the NF‐κB p65 pathway, which triggers the upregulation of proinflammatory cytokines in the colon and synovium,^16^, ^17^ as well as the AKT signalling pathway in the liver.^18^ NF‐κB is a master regulator of OA‐related inflammatory mediators and is essential to induce various proinflammatory cytokines, such as IL‐1β, IL‐6, TNF‐α and MMPs; moreover, these cytokines further activate a signalling cascade.^5^ In the present study, the NF‐κB pathway was activated in both TMJ chondrocytes and disc cells. It is well established that p65 is responsible for the transcription of target genes when the NF‐κB pathway is activated.^49^ In addition, NF‐kB is mainly activated by p65 phosphorylation and IκB kinase‐mediated IκBα degradation, while AKT can activate NF‐kB by affecting its upstream IκB kinases.^50^

As articular cartilage has a relatively simple tissue composition of only a single cell type, chondrocytes, OA pathogenesis is therefore frequently linked to changes in chondrocyte activities.^51^ The AKT pathway has received substantial attention because it plays an essential role in chondrocyte homeostasis and participates in ECM catabolism and anabolism. The AKT pathway has been reported to mediate TNF‐α expression and NF‐κB activation in osteoblasts.^52^ The AKT pathway activated in chondrocytes, plays a crucial role during several characteristic alterations of TMJOA, such as promoting the expression of MMPs directly or by activating the NF‐kB pathway. In addition, the AKT signalling pathway is a vital regulator of chondrocyte survival and apoptosis.^50^ Other research has reported that AKT pathway activation can promote chondrocyte autophagy and protect against cartilage injury.^53^ Another study showed that suppression of the AKT signalling pathway could relieve the inflammatory response in rats with OA, which means that the AKT pathway is very important during the pathogenesis of OA.^54^ In addition, the AKT pathway was not activated in chondrocytes pretreated with an inhibitor of RAGE. It has been proven that the AKT pathway regulates the expression of aggrecan, the major ECM component of the intervertebral disc, in nucleus pulposus cells.^55^ Inhibition of the AKT/NF‐κB signalling pathway ameliorated the progression of intervertebral disc degeneration (IDD).^56^ Another study demonstrated that activation of AKT suppressed degradation of ECM and inflammation, thereby alleviating IDD.^57^ The healthy intervertebral disc has few blood vessels, but some nerves are mainly restricted to the outer lamellae.^58^ TMJ discs are generally regarded as dense fibrocartilaginous discs and have no direct vascularization or nerve distribution.^9^ It was observed that the chondrocytes obtained from cartilage tissue were paving‐stone shaped or polygonal, while the disc cells obtained from disc tissue were long spindle shaped. The above complex structural differences may be the cause of the inactivation of the AKT pathway in TMJ disc cells.

In this study, it should be noted that glycyrrhizin administration downregulated the expression of RAGE and TLR4 both in vivo and in vitro. Consistent with this study, glycyrrhizin inhibited the expression of TLR4 and NF‐κB in necrotizing enterocolitis rats.^24^ Likewise, another study showed that glycyrrhizin reduced HMGB1, TLR4, IL‐1β and IL‐12 and was protective against keratitis.^22^

After applying TAK‐242 (inhibitors of TLR4) and FPS‐ZM1 (inhibitors of RAGE), MMP‐3 and IL‐6 were downregulated. In cells pretreated with TAK‐242, activation of both NF‐κB p65 and AKT was reduced; otherwise, FPS‐ZM1 downregulated the activation of NF‐κB p65. Both RAGE and TLR4 were involved in the activation of the NF‐κB p65 pathway, but only TLR4 was involved in the signal transduction of the AKT pathway. It was reported that the RAGE‐NF‐κB pathway was activated to induce inflammatory responses in human synovial cells.^17^ Meanwhile, it was shown that HMGB1 could activate TLR4‐NF‐κB signalling pathways in human intestinal epithelial cells.^16^ Another study showed that TLR4 activated the AKT pathway in tumour development.^59^ In this study, inhibition of HMGB1 receptors, whether TLR4 or RAGE, decreased the production of the cartilage degradation‐related factors MMP‐3 and IL‐6. Importantly, glycyrrhizin, like TAK‐242 and FPS‐ZM1, could reduce the expression of MMP‐3, which explained the protective effect of glycyrrhizin.

In conclusion, glycyrrhizin alleviated the pathological changes in experimental TMJOA and effectively reduced the expression of HMGB1, RAGE and TLR4. In addition, HMGB1 exerted an inflammatory and catabolic effect by activating the RAGE/TLR4‐NF‐kB p65/AKT signalling pathway to increase OA‐related factor release. This result indicated that HMGB1 acted as a signal of TMJOA inflammation, while glycyrrhizin protected the condyle from destruction.

## CONFLICT OF INTEREST

The authors declare no conflicts of interest with respect to this manuscript.

## AUTHOR CONTRIBUTIONS

**Zhihui Hu:** Conceptualization (equal); Methodology (lead); Software (equal); Writing—original draft (equal). **Mian Xiao:** Conceptualization (equal); Methodology (equal); Software (lead). **Hengxing CAI:** Conceptualization (equal); Formal analysis (lead); Software (equal). **Wei Li:** Methodology (equal). **Wei Fang:** Conceptualization (equal); Data curation (lead); Project administration (lead); Writing—original draft (equal). **Xing Long:** Conceptualization (equal); Data curation (lead); Supervision (lead); Writing—original draft (lead).

## Supporting information

Fig S1

## Data Availability

The data that support the findings of this study are available from the corresponding author upon reasonable request.
