# Supplementary figures and images for: Glycyrrhizin regulates rat TMJOA progression by inhibiting the HMGB1‐RAGE/TLR4‐NF‐κB/AKT pathway
Source: J Cell Mol Med. 2021 Dec 24;26(3):925–36. doi: 10.1111/jcmm.17149 (PMC8817133; doi:10.1111/jcmm.17149)

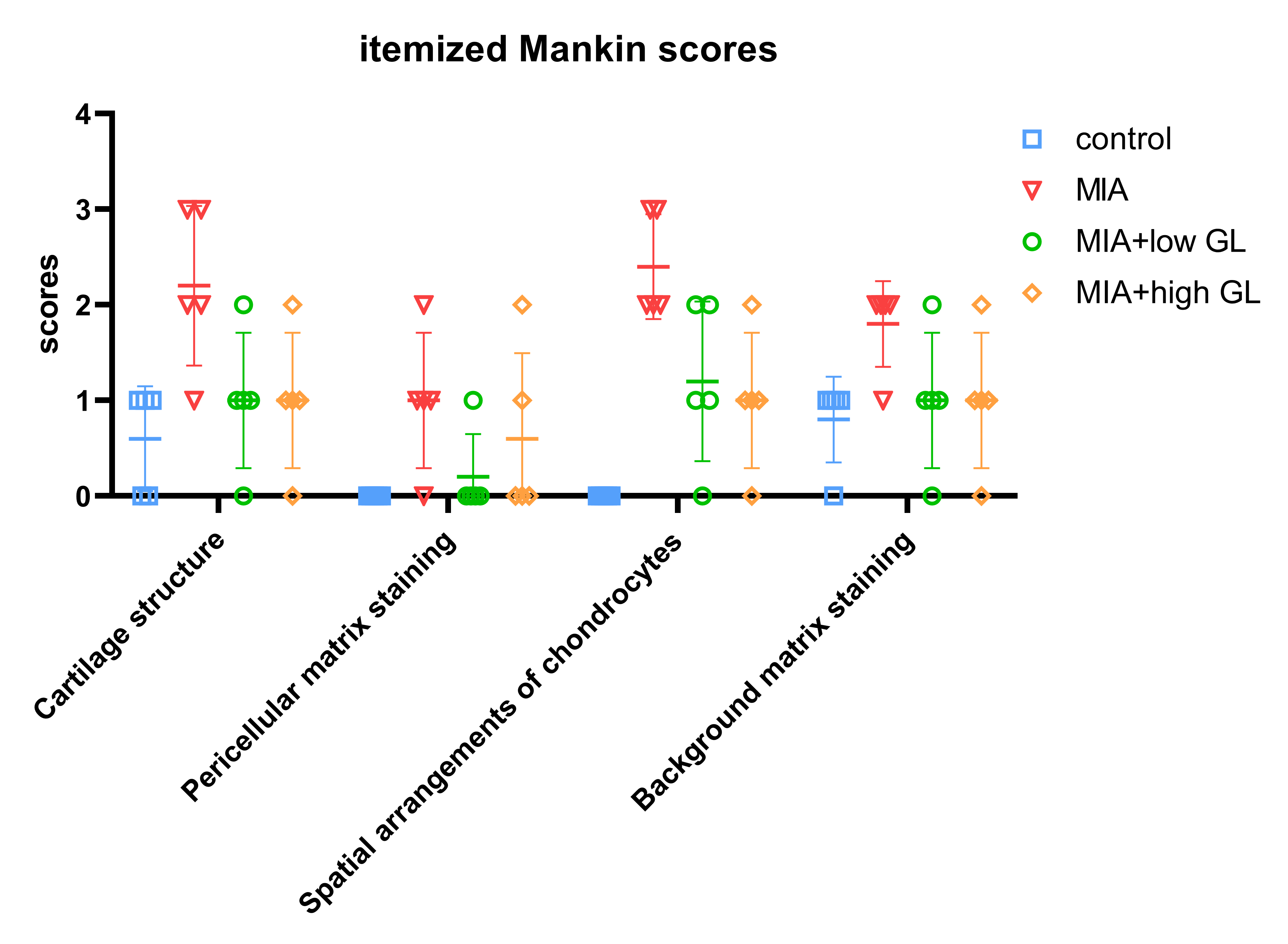

Supplement: Supplementary file 1 — Fig S1 [file JCMM-26-925-s001.png]
